# Telerehabilitation with breathing exercises as a strategy to improve functional capacity of patients with chronic obstructive pulmonary disease: randomized clinical trial

**DOI:** 10.31744/einstein_journal/2025AO1182

**Published:** 2025-11-13

**Authors:** Karine Franciele Toldo de Toledo, Sônia Maria Marques Gomes Bertolini, Sônia Trannin de Mello, Gelson Marcos Rodrigues

**Affiliations:** 1 Universidade UniCesumar Maringá PR Brazil Universidade UniCesumar, Maringá, PR, Brazil.; 2 Universidade Estadual de Maringá Maringá PR Brazil Universidade Estadual de Maringá, Maringá, PR, Brazil.; 3 Hospital Municipal de Maringá Maringá PR Brazil Hospital Municipal de Maringá, Maringá, PR, Brazil.

**Keywords:** Pulmonary disease, chronic obstructive, Rehabilitation, Exercise tolerance, Functional status, Telerehabilitation

## Abstract

**Objective:**

This study aimed to analyze the effects of two proposed telerehabilitation interventions on the functional capacity of patients with chronic obstructive pulmonary disease.

**Methods:**

A randomized clinical trial was conducted with 60 patients of both sexes equally distributed into two groups: G1, submitted to a protocol of breathing exercises and health education; and G2, submitted only to health education. The interventions were performed remotely through videoconferencing with patients in their homes for 8 weeks.

**Results:**

When comparing the pre- and post-intervention moments of G1 and G2, significant differences were found in the functional capacity scores in both cases (p=0.0001), however, when comparing the post-intervention moment of the two groups, the improvement was more significant in G1 (p=0.0007).

**Conclusion:**

Both telerehabilitation with a respiratory exercise protocol associated with health education, and telerehabilitation with only a health education protocol are beneficial for the functional capacity of patients with chronic obstructive pulmonary disease. However, the telerehabilitation with a breathing exercise protocol associated with health education had a greater impact on the improvement of patients with chronic obstructive pulmonary disease.

## Highlights

Telerehabilitation included breathing exercises and health education.

Health education alone was applied in the Control Group.

Remote sessions were conducted at home via videoconference for 8 weeks.

Breathing exercises improved functional capacity in patients with chronic obstructive pulmonary disease.

## In Brief

This study analyzed the effects of two telerehabilitation interventions on functional capacity in patients with chronic obstructive pulmonary disease. The combination of breathing exercises and health education significantly improved the outcomes, offering a feasible and effective alternative for expanding access to pulmonary rehabilitation.

## Graphical abstract

**Figure:**
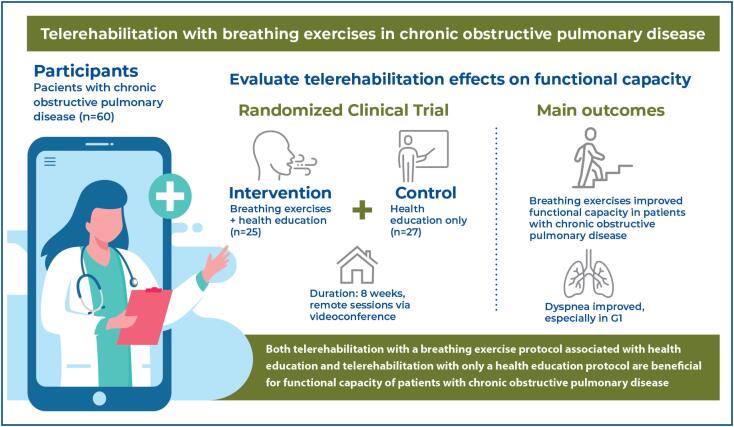

## INTRODUCTION

Chronic obstructive pulmonary disease (COPD) is a complex and heterogeneous condition that poses a significant challenge to public health worldwide.^(1,2)^ It is characterized by the irreversible and progressive obstruction of airflow due to changes in the airways and pulmonary alveoli resulting from inflammatory and structural interactions. These changes contribute to lung damage thereby affecting their development and aging processes.^(3,4)^

In addition to its significant health impacts, COPD imposes a substantial burden on global morbidity and mortality rates.^(5,6)^ In Brazil, its prevalence is remarkable, especially in adults over 60 years of age, reflecting the influence of smoking and air pollution.^(7-9)^ The prevalence of COPD is expected to increase due to continuous exposure to risk factors and aging population.^(10)^

The symptoms of COPD are varied including dyspnea, fatigue, and sleep disorders, which significantly affect the quality of life.^(11,12)^ Thus, management of such condition requires a multidisciplinary approach, with Pulmonary Rehabilitation (PR) playing a fundamental role.^(13,14)^ However, despite the benefits of PR, there are significant challenges in participating in in-person programs, such as barriers to access and adherence.^(15)^

In this context, telerehabilitation has emerged as a promising alternative, offering remote access to PR services, including breathing exercises, and demonstrating similar results to traditional PR.^(16)^ Breathing exercises are essential components of PR as they improve respiratory muscle function and reduce symptoms.^(17,18)^ These exercises include techniques such as diaphragmatic- and pursed-lip- breathing, which have significant benefits on lung function and quality of life.^(19,20)^

The findings are expected to contribute to a better understanding of the potential benefits of telerehabilitation in the management of COPD and provide important insights into clinical practice and public health policies related to the treatment of this chronic condition.

## OBJECTIVE

In this study, we aimed to analyze the effects of two proposed telerehabilitation interventions on the functional capacity of patients with chronic obstructive pulmonary disease.

## METHODS

### Study design

For this randomized parallel-group clinical trial, 86 patients who met the inclusion criteria were considered eligible to participate in the study. Of these, 21 were excluded; six did not agree to participate in the study, and 15 presented reasons, such as lack of time for their non-participation. However, of these 65 eligible patients, only 60 presented themselves for initial assessment, and signed the Free and Informed Consent Form. The participants were then randomized and distributed equally into two groups: G1 – to undergo telerehabilitation with a breathing exercise protocol and Health education; and G2 – submitted to telerehabilitation only with a health education protocol (n=30 in each group).

**Figure 1:**
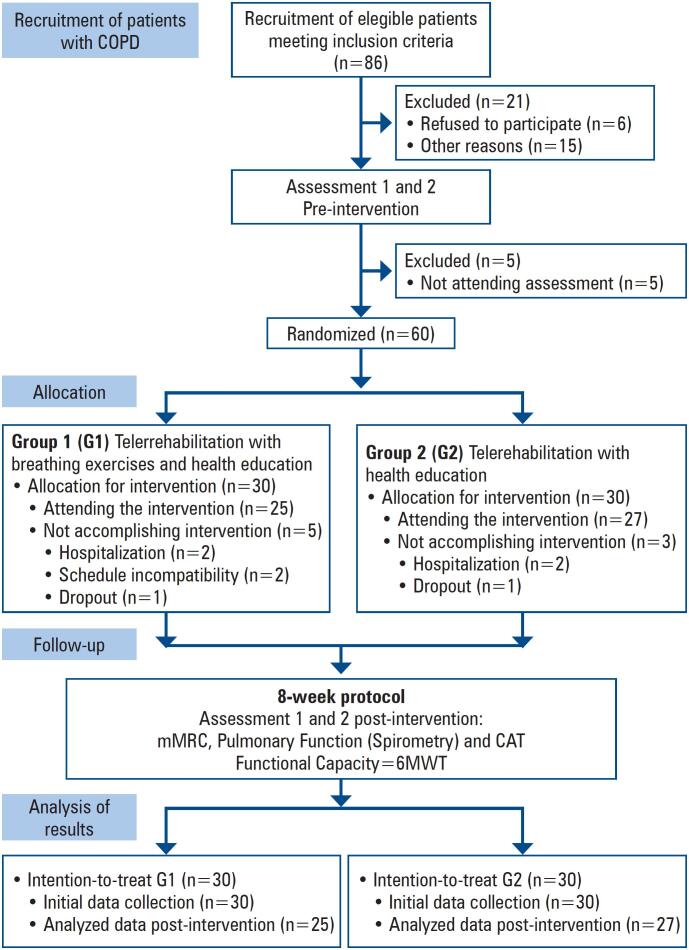
Randomized clinical trial design

COPD: chronic obstructive pulmonary disease; mMRC: modified Medical Research Council dyspnea scale; CAT: COPD Assessment Test; 6 MWT: 6-min walk test.

After allocation to intervention groups, during the protocol development period, there was a loss of five patients allocated to G1 and three patients allocated to G2. Both groups were evaluated before and after the intervention. The study design is shown in figure 1 which was prepared in accordance with the recommendations of the Consolidated Standards of Reporting Trials (CONSORT).^(21,22)^

### Participants

The present study was carried out with a sample initially consisting of 60 patients who presented themselves for consultation at a pulmonology outpatient clinic in the city of Maringá-PR during the study period (September 2023 to December 2023). Patients included were of both sexes, aged between 58 and 64 years old, with a clinical diagnosis of COPD, classified according to the guidelines of the Global Initiative for Chronic Obstructive Lung Disease,^(1)^ who had not participated in a PR program in the last six months before the beginning of the intervention and were clinically stable (without exacerbations and/or hospitalization in the last 4 weeks).

Patients with any other serious or debilitating condition, who were unable to comprehend and/or perform the study procedures, or who had a compromised general condition that made their participation impossible were excluded.

Participants were approached and invited to participate in the study by the researchers, and the study began only after explaining the objectives of the study, reading out the Free and Informed Consent Form, and obtaining their signed consent.

This study was approved by the Research Ethics Committee of *Universidade UniCesumar*, CAAE: 73290923.1.0000.5539; # 6,271,954.

### Allocation and randomization

Patients were randomized to two telerehabilitation groups: Group 1 (G1), in which the participants underwent a protocol of breathing exercises and health education sessions, and Group 2 (G2), in which they received only health education sessions. The intervention protocol for patients in G1 was offered to patients in G2, after the end of the study period. The allocation sequence was generated using a random table created at www.randomizer.org.

### Blinding procedure

All evaluators remained blinded to the group allocation, hypotheses, and intervention details. It was not possible to hide the allocation of the therapist and participants, as the breathing exercise interventions were performed. The professional who performed the statistical analyses was blinded to the group allocation.

### Data collection

Data were collected only when the eligibility criteria for the study was fulfilled. The same researcher evaluated both groups before and after the intervention. Two evaluators were trained by the lead researcher for data collection.

The pre-intervention assessment was performed twice, with an interval of two–three days between them. The first stage of the pre-intervention assessment was called AS1 and comprised the application of the sociodemographic and health questionnaire, modified Medical Research Council dyspnea scale (mMRC)^(23)^ and peripheral oxygen saturation (SpO2) after 5 min of rest (G-TECH oximeter model Oled Graph, MD300C23). The second stage of the pre-intervention assessment (AS2) included spirometry, the 6-min walk test, and application of the COPD Assessment Test (CAT) questionnaire to assess the clinical impact of COPD.

The sociodemographic and health questionnaire consisted of the following variables: age, sex, self-reported skin color, marital status, years of schooling, time of COPD diagnosis in years, length of outpatient follow-up in years, smoking status, smoking load, use of inhaled medications for the treatment of COPD, oxygen dependence, other associated comorbidities, visits to emergency services or hospitalizations for COPD in the last 12 months, participation in a pulmonary rehabilitation (PR) program, and physical activity.

Participants underwent spirometry (Spirolab^®^ spirometer from Medical International Research, Rome, Italy) in accordance with the standards of the American Thoracic Society/European Respiratory Society.^(24)^ The spirometric and multidimensional classification of the Global Initiative for Chronic Obstructive Long Disease (GOLD) 2024 was used to stratify the severity of COPD according to the degree of obstruction to expiratory flow, FEV1 (forced expiratory volume in the 1st second), according to the which, FEV1 ≥80% of predicted was considered as mild COPD (GOLD 1), 50 ≤FEV1 <80% of predicted was considered as moderate COPD (GOLD 2); 30 ≤FEV1 <50% of predicted, as severe (GOLD 3); and FEV1 <30%, as very severe (GOLD 4).^(1)^

The clinical impact of COPD was classified based on the score obtained on the COPD Assessment Test (CAT), which has been validated in Portuguese for use in Brazil. This questionnaire consisted of eight items: cough, phlegm, chest tightness, exertional dyspnea, activities of daily living, confidence, sleep, and energy. For each item, the patient chose only one answer, and the score ranged from 0 to 5. At the end of the test, the scores of all responses were summed; thus, the clinical impact of COPD was assessed according to score stratification. The results were classified in relation to the clinical impact as follows: 6-10 points, mild; 11-20, moderate; 21-30, severe; and 31-40, extremely severe.^(25)^

The GOLD proposes a combined assessment strategy for airflow limitation (GOLD grades 1-4) with the level of symptoms (mMRC and CAT) and history of previous exacerbations, defined as classifications A, B, and E. This classification incorporates patient-reported outcomes and highlights the importance of preventing exacerbations in the treatment of COPD, where group A is defined as mMRC 0-1, CAT <10 and none or one moderate exacerbation without requiring hospitalization per year; group B is defined as mMRC ≥2, CAT ≥10 and none or one moderate exacerbation not requiring hospitalization per year; and group E ≥2 moderate exacerbations per year or ≥1 exacerbation requiring hospitalization.^(1)^

To assess functional capacity, the 6-min walk test (6 MWT) was performed according to the American Thoracic Society guidelines.^(24)^ After the 6 MWT, the distance walked in meters (6 MWD) was calculated and recorded. For the primary outcome of the study, a change of 26m between the initial and final assessments was considered the minimum clinically relevant difference, according to data from previous studies, with a confidence level of 80%.^(26,27)^ However, for 95% confidence level, indicating that the improvement was clinically significant, an increase >70m in the 6 MWD after the intervention was considered.^(24)^

Participants were reassessed after the intervention period using the assessments described previously (AS1 and AS2), which were conducted by the same evaluator who performed the pre-intervention assessment.

### Intervention

After the assessments, patients were randomized using a randomization table. After performing the physical tests on the day of AS2, the participants allocated to G1 received supervised in-person training on the proposed breathing exercise protocol. The intervention for G1 was continued remotely via videoconference with patients in their homes.

The intervention was supervised. Each session lasted approximately 20 min, with 15 min of breathing exercises and 5 min of daily assessment, carried out using an online questionnaire on Google Forms, consisting of the modified Borg scale that assesses the sensation of dyspnea (0–10 points, 0, no dyspnea; 10, maximum dyspnea)^(28)^ presence of pain assessed by the visual analog scale (VAS), presence of fever, or other changes/discomfort. The sessions were held five times a week over a period of eight weeks, taking place at a predetermined time (between 6:30 pm and 9:30 pm) after contacting the researcher via video call; 10% absenteeism was accepted during the intervention period.

The breathing exercises used in the protocol were identified and selected from exercises tested in previous intervention studies in patients with COPD. ^(18)^ The proposed breathing exercise protocol consisted of performing diaphragmatic exercises associated with pursed lips for 15 min. Exercise was monitored by a researcher. The patients were instructed to position their smartphones in such a way that it was possible for the researcher to visualize the exercises being carried out correctly. Verbal instructions for performing the exercises were repeated and reinforced during the execution.

Patients were instructed to remain seated with the spine supported comfortably in an armchair, chair, or sofa. They were asked to concentrate on their respiratory cycle (inspiration and exhalation), and then a verbal command was given to perform the breathing exercise with the therapist controlling the time with a stopwatch. Breathing exercises were performed for 15 min with a frequency of eight–ten repetitions per series. Between series, the patient was allowed to take calm, uncontrolled breaths.

To perform diaphragmatic exercises, patients were instructed to perform nasal inspiration, predominantly by moving the abdomen and reducing the movement of the rib cage. For diaphragmatic breathing in conjunction with pursed-lip breathing, patients were instructed to perform diaphragmatic breathing and then exhale air slowly with the lips partially closed. Standardized verbal instructions were given to ensure correct performance of the technique, which was taught on the day of AS2, with the therapist making appropriate corrections when necessary. The learning process was reinforced during video-call sessions.

Health education sessions were offered to both groups (G1 and G2), for which eight meetings were held. Patients (maximum 6) participated once a week, remotely via videoconference, in sessions lasting 24–27 min. The topics covered in the educational program included the concept of COPD and treatment; the importance of stopping smoking; the importance of daily physical activity and exercise; early signs of COPD exacerbation; emotional, psychological, and social well-being in patients with COPD; sleep quality in COPD; quality of life with COPD; and aging with COPD. A printed booklet with supporting content for the meetings was delivered on the day of AS2. Participants were informed about meetings, and a link to the video conference was sent weekly via WhatsApp on a previously agreed day and time. The topics were promoted as a combination of information, dialogue, reflection exercises, focus on increasing self-competence, and exchange of experiences, all with the aim of communication and learning. The health education program was based on an interventional study involving patients with COPD.^(29)^

### Statistical analysis

Data were described using descriptive measures and frequency tables. To compare the difference in the spirometry test observations, the paired *t*-Student test for comparison within groups, and the Student *t*-test for independent data for comparison between groups, was applied. For the Likert scales, equivalent non-parametric tests were used: the Wilcoxon sign test (paired) and the Wilcoxon test for independent data. Data abnormalities were tested using the Shapiro-Wilk test. Data were analyzed using SAS software (Statistical Analysis Software) version 9.4. A confidence level of 95% was considered as statistically significant.

## RESULTS

### Recruitment and baseline characteristics

A total of 60 patients were randomized to groups G1 and G2; however, at the beginning of the intervention, five patients allocated to G1 and three allocated to G2 were lost. During the protocol application period, even after the losses, no significant changes were observed in the structure of the groups with respect to sex (p=0.1217) or education (p=0.1323).

The age of the patients ranged from 58 to 63 years (mean=60.64 and SD=1.08) in G1, and from 60 to 64 years (mean=60.81 and SD=0.921) in G2. In G1, the majority (60.00%) of patients were diagnosed with COPD within five years, whereas in G2, the majority (59.26%) of patients were diagnosed with COPD more than five years ago. More than half of the patients in G1 (56.00%) and G2 (55.56%) were smokers. However, the number of patients with a smoking history of >40 pack-years was higher in G1 (52.00%). The number of patients having some type of comorbidity was similar in G1 (76.00%) and G2 (74.07%). In the last 12 months, all patients in G1 and G2 required emergency services/hospitalization because of COPD. Only one patient from G2 sought treatment twice and required hospitalization. None of the patients had participated in PR programs. The baseline characteristics of the patients are shown in table 1.

**Table 1:** Baseline characteristics of study participants

Variable		G1 (n=25)	G2 (n=27)	p value
Gender, n (%)				
	Female	11 (44.00)	16 (59.26)	0.1217
	Male	14 (56.00)	11 (40.74)	
Age (years), mean (SD)		60.64 (1.08)	60.81 (0.921)	0.5310
Formal education, n (%)				
	Up to 8 years	10 (40.00)	7 (25.93)	0.1323
	Over 8 years	15 (60.00)	20 (74.07)	
Comorbidity, n (%)				
	Yes	6 (24.00)	7 (25.93)	0.2477
	No	19 (76.00)	20 (74.07)	
Smoking status, n (%)				
	Former smoker	11 (44.00)	12 (44.44)	0.2196
	Active smoker	14 (56.00)	15 (55.56)	
Smoking history (pack-year), mean (SD)		45.60 (14.02)	45.37 (13.44)	0.9522
Exacerbations in the last 12 months, n (%)		25 (100)	27 (100)	-
FEV1, % predicted, post-BD, mean (SD)		75.28 (9.94)	74.30 (11.19)	0.7386
FEV1/FVC (%) post-BD, mean (SD)		65.44 (2.72)	65.89 (2.45)	0.5348
Gold, n (%)				
	1 mild	7 (28.00)	8 (28.00)	
	2 moderate	17 (68.00)	17 (62.96)	0.8506*
	3 severe	1 (28.00)	2 (7.41)	
	4 very severe	0	0	
COPD classification in relation to history of exacerbations and symptoms (CAT and mMRC), n (%)				
	A	7 (28.00)	8 (29.63)	
	B	17 (68.00)	17 (62.96)	0.8506*
	E	1 (4.00)	2 (7.41)	
Classification of COPD in relation to clinical impact (CAT), n (%)			0	
	Mild	7 (28.00)	8 (29.63)	
	Moderate	17 (68.00)	17 (62.96)	0.8506*
	Severe	1 (4.00)	2 (7.41)	
	Extremely severe	0	0	
mMRc, mean (SD)		1.76 (0.52)	1.78 (0.58)	0.9081
SpO2 (%) in resting, mean (SD)		90.68 (1.44)	90.48 (1.40)	0.6156

* Significant at 95% confidence level.

COPD: chronic obstructive pulmonary disease; GOLD: Global Initiative for Chronic Obstructive Lung Disease; FEV1: forced expiratory volume in the 1st second; FEV1/FVC: forced expiratory volume in 1st second/forced vital capacity; mMRC: modified Medical Research Council dyspnea scale; CAT: COPD Assessment Test; SpO2: peripheral oxygen saturation.

No change was observed in the classification of COPD severity according to GOLD criteria in either group. However, after the intervention, the clinical impact of COPD, classified based on the CAT, showed a change from moderate to mild (Table 2).

**Table 2:** Severity of chronic obstructive pulmonary disease in patients

Severity of COPD		Before			After		
		G1	G2	p value	G1	G2	p value
Gold							
	1 mild	7 (28.00)	8 (29.63)		7 (28.00)	8 (29.63)	
	2 moderate	17 (68.00)	17 (62.96)	0.8506	17 (68.00)	17 (62.96)	0.8506
	3 severe	1 (4.00)	2 (7.41)		1 (4.00)	2 (7.41)	
CAT							
	Mild	7 (28.00)	8 (29.63)		15 (60.00)	13 (48.15)	
	Moderate	17 (68.00)	17 (62.96)	0.8506	9 (36.00)	12 (44.44)	0.6606
	Severe	1 (4.00)	2 (7.41)		1 (4.00)	2 (7.41)	

COPD: chronic obstructive pulmonary disease; GOLD: Global Initiative for Chronic Obstructive Lung Disease; CAT: COPD Assessment Test.

To test for differences in the spirometry parameters between G1 and G2, post-intervention measurements were considered. After applying the telerehabilitation protocol, no significant differences were observed between the groups (Table 3).

**Table 3:** Spirometric characteristics of study participants

Spirometry	After telerehabilitation				p value
	G1		G2		
	Mean	SD	Mean	SD	
FEV_1_	1.99	0.39	2.00	0.41	0.9394
FEV_1_ predicted (%)	74.12	9.73	73.15	11.20	0.7406
FVC	2.47	0.39	2.48	0.42	0.9633
FEV_1_/FVC	65.44	2.72	65.89	2.45	0.5348

COPD: chronic obstructive pulmonary disease; FEV1: forced expiratory volume in the 1st second; FVC: forced vital capacity; FEV1/FVC: forced expiratory volume in 1st second/forced vital capacity.

### Primary outcome

#### Functional capacity

In both groups, an improvement in the functional capacity of the patients was observed with a change in the distance covered during the 6 MWT. Table 4 shows the intergroup differences at the end of the intervention protocol. The distributions of score differences within both groups were not normal (Shapiro-Wilk ≤0.05). Differences were tested using a paired Wilcoxon test. After the intervention, both groups showed a statistically significant improvement in functional capacity.

**Table 4:** 6 MWT scores of study participants

		Mean	SD	p value
Group 1				
	6MWD pre-intervention	307.12	34.23	0,0001*
	6MWD post-intervention	363.40	33.23	
Group 2				
	6MWD pre-intervention	305.70	38.00	0,0001*
	6MWD post-intervention	323.00	40.79	

* Significant at 95% confidence level.

COPD: chronic obstructive pulmonary disease; 6 MWD: walking distance in meters in the 6 MWT.

Although both groups demonstrated improvements in functional capacity after the intervention, the gain was greater in G1 as compared to that in G2. Patients in G1 covered an average of 40 m more than those in G2, with a statistically significant difference (Table 5).

**Table 5:** 6-min walk test (6MWT) of study participants

6MWD	After intervention						p value
	G1			G2			
	Mean	SD	Average score	Mean	SD	Average score	
	363.40	33.23	33.54	323.00	40.79	19.98	0.0007*

* Significant at 95% confidence level.

COPD: chronic obstructive pulmonary disease; 6 MWD: walking distance in meters in the 6 MWT.

### Secondary outcomes

#### Effect of the telerehabilitation protocol on peripheral oxygen saturation (SpO2) and dyspnea

Patients allocated to either G1 or G2 showed an increase in peripheral oxygen saturation after eight weeks of telerehabilitation intervention (Table 6).

**Table 6:** SpO2 (%) of study participants

		Mean	SD	p value
Group 1				
	SpO2 before	90.68	1.44	0.0001*
	SpO2 after	93.04	1.06	
Group 2				
	SpO2 before	90.48	1.40	0.0001*
	SpO2 after	93.11	1.37	

* Significant at 95% confidence level.

COPD: chronic obstructive pulmonary disease, SpO2: peripheral oxygen saturation.

The findings have a normal distribution (Shapiro-Wilk ≥0.05), therefore differences in means were tested within each group using the paired *t*-Student test and between groups using the *t*-Student test for independent data. In both groups, a significant difference (p=0.0001) was observed in the SpO2. No significant difference (p=0.8358) was observed in the mean blood oxygen saturation between the groups.

In both groups, the mean mMRC dyspnea scale scores decreased after eight weeks of intervention, suggesting an improvement in the sensation of shortness of breath; however, only Group 2 showed a significant difference (p=0.002) in the degree of dyspnea. No significant difference (p=0.1403) was observed in the mean degree of dyspnea between the groups (Table 7).

**Table 7:** mMRC of study participants

		Mean	SD	p value
Group 1				
	mMRC before	1.76	0.52	0.0625
	mMRC after	1.56	0.58	
Group 2				
	mMRC before	1.78	0.51	0.0020*
	mMRC after	1.41	0.50	

* Significant at 95% confidence level.

COPD: chronic obstructive pulmonary disease; mMRC: modified Medical Research Council Dyspnea Scale.

Regarding peripheral oxygen saturation and dyspnea monitored daily using the Borg scale before and after performing the breathing exercise protocol in G1, the data presented a normal distribution (Shapiro-Wilk≥0.05), therefore the differences in the mean were tested by using the paired *t*-Student test. Immediately after the exercises, patients in G1 showed significantly improved SpO2 and reduced dyspnea intensity (p=0.0001) (Table 8).

**Table 8:** SpO2 (%) and Borg Scale of study participants

Statistics	G1	
	SpO_2_	Borg
Mean difference	3.13	3.52
Standard deviation	1.22	0.87
Median	3.00	3.50
Normality test (Shapiro-Wilk)	0.0561	0.0778
t-Student (paired)	0.0001*	0.0001*

* Significant at 95% confidence level.

COPD: chronic obstructive pulmonary disease, SpO2: peripheral oxygen saturation.

## DISCUSSION

In this study, a randomized clinical trial was carried out in patients with COPD. A comparative study was conducted by submitting the patients to a telerehabilitation program consisting of either breathing exercises combined with health education (G1), or health education alone (G2). The trial was designed to provide preliminary evidence for the use of telerehabilitation by analyzing functional capacity performance with the 6 MWT as the primary outcome. The results support the hypothesis that in both groups, there was an increase in the distance walked in the 6 MWT, with G1 being superior to G2.

Furthermore, no difference was observed between the groups in relation to the validated clinical score for feeling shortness of breath (modified Medical Research Council dyspnea scale - mMRC) after the eight-week intervention period. However, immediately after the breathing exercises carried out by G1, there was an improvement in the sensation of dyspnea, verified using the Borg scale, and an improvement in SpO2 with a statistically significant difference, demonstrating the benefit of the telerehabilitation protocol carried out with breathing exercises.

PR is part of the standard treatment for patients with COPD who are functionally limited, and telerehabilitation has been shown to be an option for PR, presenting similar benefits to those of in-person PR, being able to offer health care through the use of telecommunications and virtual technology.^(15)^

The impact of PR and telerehabilitation was analyzed in a randomized clinical trial,^(30)^ in which 90 patients with COPD, with a mean age of 70 years, were randomized into conventional in-person PR, and online PR for six weeks. The distance walked during the 6 MWT increased in both groups after the intervention. The adjusted mean difference in the 6 MWT between the groups for the population did not demonstrate inferiority of the online intervention.^(30)^ These findings corroborate the present study, which showed improvements in functional capacity in both groups irrespective of performing breathing exercises. Thus, the importance of promoting health education is evident. Furthermore, health education is a recognized and important component of PR, as it helps promote self-management and knowledge about the disease, and can change outcomes in patients with COPD.^(1)^

In a randomized study carried out with a home PR model using minimal resources and little direct supervision, short-term improvements in the 6 MWD were verified, and the results were found to be similar to those of an in-person rehabilitation program. The study included 166 patients with stable COPD, with an average age of 69 years. Participants were randomly distributed into two groups: one group in person and the other group at home; and underwent eight weeks of PR. Statistical analysis confirmed the non-inferiority of home-based rehabilitation for the 6 MWT at the end of rehabilitation. As a secondary outcome, the differences between the groups in quality of life related to dyspnea were analyzed. Results showed that the groups were equivalent in terms of quality of life 12 months after intervention.^(31)^ These findings are similar to those in the present study; therefore, home PR may be considered for people with COPD who do not have access to PR in healthcare facilities, such as physiotherapy clinics and rehabilitation centers.

Although evidence for the safety and benefit of PR in patients with stable COPD is well established, evidence regarding the best exercises and intervention modalities is not as strong. The current design and delivery of educational and exercise interventions are largely based on best practices. Telerehabilitation models have the potential to positively influence the acceptance and accessibility of PR services for all patients with COPD.^(15)^

Recent guidelines on PR emphasize that the ideal structure of PR remains unknown, requiring more studies to determine the quality, cost-benefit, and accessibility to public health policies, highlighting technologies in the investigative process.^(32)^

Despite the widespread use of online technologies to manage almost every aspect of daily life, few well-conducted clinical trials have been reported in the management of COPD. After the COVID-19 pandemic, telerehabilitation became evident as an alternative for patients with indications for PR, and was considered the most appropriate method as it allowed social distancing and decreased the risk of transmission. This alternative also proved to be as beneficial as conventional PR in patients with chronic respiratory diseases, and presented itself as a favorable cost-benefit model.^(33)^ The approach of the present study offers new evidence that telerehabilitation may benefit patients with COPD who can participate in PR through the use of devices such as smartphones, which are widely used.

It is imperative to implement a coordinated approach to stimulate overall health improvement with a focus on the increasing use of mobile services and emerging health technologies. However, this requires a multidisciplinary endeavor to ensure the formulation of a patient-centered coordinated strategy accompanied by tests to establish clinical benefits and cost-effectiveness.^(34)^

Limited resources, geographic distance, and the availability of appointments compromise the provision of in-person PR.^(35)^ In the present study, all patients had access to the Internet, which was an eligibility criterion. Reports during weekly health education sessions demonstrate that the alternative to remotely accessing healthcare is accepted and is looked upon as a great opportunity to improve health. Patients reported on several occasions that the chance to talk about their disease, ask questions, and reflect on the subject served as an incentive to improve aspects crucial to their quality of life and living with COPD.

Regarding the better results observed in G1, it is believed that holding daily meetings (five times a week) resulted in participants gaining greater motivation and incentives to improve the determining aspects of the health-disease process. Some participants reported being more willing to start physical activities and stop smoking.

In the context of aging, it is widely recognized that several physiological changes can negatively impact walking ability and functionality of individuals.^(36)^ However, the results of this study provide encouraging evidence that even with adversity, it is possible to achieve significant improvements, such as an increase in the 6 MWD in older adults. Finally, individual-centered intervention strategies are important to optimize the functional capacity of a specific population.

### Study limitations

The present study outlined an investigation of the effects of telerehabilitation on functional capacity of patients with COPD, through a randomized clinical trial of parallel groups. Although meticulously designed, this study has some limitations that must be considered.

The eight-week intervention period evaluated the immediate effects of telerehabilitation, not allowing inferences about the sustainability of these effects in the long term. Even if the results provide valuable insights into the effects of telerehabilitation in patients with COPD, it is essential to analyze them with caution, taking into account the limitations concerning the design and sample size.

The lack of part of the study involving only breathing exercises also need to be highlighted. Future studies should expand the sample size, strengthen the obtained results, and randomize participants into four groups: Group 1, breathing exercises and health education; Group 2, health education; Group 3, breathing exercises; and Group 4, controls (without intervention). It is also suggested to extend the follow-up period after developing the protocol and check whether the benefits can be sustained. In addition, there is a need for research to determine the best telerehabilitation models, their cost-benefit ratio and long-term impact on COPD management.

Despite these encouraging results, it is important to emphasize that there were technical challenges related to the use of technology by participants such as handling and positioning smartphones during video calls. Nevertheless, the role of technology in supporting self-management in patients with COPD should be addressed and encouraged in future clinical studies.

### Applicability

The structured home telerehabilitation approach proved to be effective in improving functional capacity and quality of life related to dyspnea, especially with breathing exercises combined with health education, and was demonstrated to be a viable and effective option for patients with COPD. Therefore, it is recommended that healthcare professionals consider implementing telerehabilitation programs into their clinical practices to expand access to PR. It is also important to integrate digital approaches and promote a multidisciplinary culture to ensure general improvements in the health and quality of life of patients with COPD. In this study, significant potential of telerehabilitation for improving access to healthcare for patients with COPD is suggested, and their acceptance and accessibility have been highlighted.

Our findings may also contribute to cost reduction within the healthcare system, in addition to mitigating logistical challenges or transportation barriers that prevent in-person treatment.

## CONCLUSION

This study demonstrated that both telerehabilitation interventions, one with a breathing exercise protocol associated with health education and the other with only a health education protocol, are beneficial for functional capacity of patients with chronic obstructive pulmonary disease. However, the impact of telerehabilitation with a breathing exercise protocol associated with health education tends to be more effective for patients with chronic obstructive pulmonary disease as demonstrated by their ability to walk a greater distance in the 6 MWT.
